# Natural and Regenerated Cellulosic Microfibers Dominate Anthropogenic Particles Ingested by Commercial Fish Species from the Adriatic Sea

**DOI:** 10.3390/foods14071237

**Published:** 2025-04-01

**Authors:** Serena Santonicola, Michela Volgare, Federico Olivieri, Mariacristina Cocca, Giampaolo Colavita

**Affiliations:** 1 Department of Medicine and Health Sciences “V. Tiberio”, University of Molise, 86100 Campobasso, Italy; serena.santonicola@unimol.it (S.S.); colavita@unimol.it (G.C.); 2 Institute of Polymers, Composites and Biomaterials, National Research Council of Italy, Via Campi Flegrei 34, 80078 Pozzuoli, Italy; federico.olivieri@cnr.it; 3 Department of Chemical Engineering, Materials, and Industrial Production, University of Naples Federico II, P. Tecchio 80, 80125 Naples, Italy; michela.volgare@unina.it

**Keywords:** cellulosic microfibers, microplastics, commercial fish, Adriatic Sea

## Abstract

This study investigated the occurrence of fibrous microplastics and natural and artificial cellulose microfibers in the gastrointestinal tracts of *Mullus barbatus* and *Merluccius merluccius* specimens from the Adriatic Sea (Central Mediterranean), an important hotspot for marine litter accumulation. Red mullet and European hake were chosen due to their roles as bioindicators of marine pollution in the Mediterranean, and their economic relevance as fishery resources. Microfibers were found in 72% of *M. barbatus* and 68% of *M. merluccius*, at levels ranging from 1 to 67 particles/individual. Most of the microfibers extracted were textile fibers that were blue (33.6%), clear (26.1%), and black (20.3%) in color, while the length distribution showed the prevalence of microfibers in the size range of 350–950 µm. This visual identification, corroborated by the micro-FTIR analysis of a sub-sample of microfibers, revealed that natural and artificial cellulose microfibers were more common (80%) than fibrous microplastics. The results confirmed that both of these fish species are susceptible to microfiber ingestion and indicated the high availability of natural and artificial cellulosic fibers in the Adriatic Basin. Despite the increased evidence of microfiber pollution in the marine ecosystem, only a limited number of studies examine natural/artificial microfiber contamination and ingestion by marine biota. Therefore, greater attention should be given to this new type of contaminant, considering its implications in terms of environmental health, food security, and food safety.

## 1. Introduction

Recently, among the emergent pollutants in the aquatic ecosystem, growing research attention is being focused on natural and regenerated cellulosic microfibers as an important form of marine contamination [1,2,3].

Microfibers have been defined as “any natural or artificial fibrous materials of threadlike structure with a diameter less than 50 μm, and length ranging from 1 μm to 5 mm” [1]. Natural microfibers can be derived from natural fibers such as cotton, flax, wool, and silk, while fibrous microplastics may be produced by synthetic fibers such as nylon, polyester, polyolefin, and acrylic. Artificial microfibers can be released from regenerated cellulosic fibers (i.e., derived from the chemical transformation of natural materials), such as viscose or rayon. These last are not considered microplastics because of their non-petroleum origin [4].

Fibrous materials of sub-millimetric size represent widespread pollutants in the environment, as these particles may be released during the washing of clothes or may be produced by the textile industry and shed from upholstery, carpets, and other textiles [5,6]. Synthetic microfibers released from the clothes chain are a prevalent type of microplastics in the marine environment [7]. However, recently, it has been found that natural and artificial textile microfibers constitute the most important fraction of microfibers in different environments compared with synthetic ones [6,8,9]. Half of the anthropogenic debris in marine sediments are rayon fibers [10], and up to 79.5% of the microfibers in water samples are cellulosic fibers [9]. Despite these findings, few studies are focused on the occurrence, prevalence, and impacts of natural and regenerated cellulose microfibers in marine biota [11]. Due to analytical limitations in terms of microfiber isolation and chemical characterization from biological samples, these particles were often excluded or incompletely described in the literature, underestimating the real level of contamination [7]. In this context, researchers have been encouraged to assess the extent of microfiber exposure in the organisms inhabiting aquatic environments separately from the microplastic problem, taking into account the different environmental abundance and ecotoxicological effects [2,12]. Microfibers seem to be more toxic for marine organisms than spheres and fragments of the same polymer [11]. Moreover, they may contain numerous additives and chemical compounds that potentially harm the marine biota and fish species intended for human consumption, leading to human exposure [4].

Due to their high bioavailability, microfibers have been detected in the gastrointestinal tract (GIT) of numerous marine organisms, including edible fish and shellfish, as the predominant component of ingested debris [3,7,12,13,14]. Consequently, the harmful effects related to microfiber exposure would result in risks to food security in the near future. Moreover, seafood contamination represents one of the major pathways of microfibers into humans, also posing a potential threat in terms of food safety. However, there is still insufficient awareness of the extent of microfiber exposure in commercial fish species intended for human consumption; thus, at the moment, it is not possible to perform a risk assessment of human exposure [15,16].

The different habitats and feeding modes of fish species may contribute to their susceptibility to microfiber ingestion, with benthic fishes being more exposed [17]. One plausible explanation is that cellulose microfibers, being denser than water, may sink to deep-sea sediments [18], making them available for benthic feeders [14]. A previous survey showed the presence of microfibers in the GITs of pelagic fishes, but higher levels and greater frequency of ingestion were detected in the demersal red mullet (*Mullus barbatus*) and benthopelagic European hake (*Merluccius merluccius*) collected from the same fishing area (Western Mediterranean Sea). This assessment of the potential effect of fiber ingestion on exposed exemplars highlighted that microfibers may affect their health status [19].

Therefore, the aim of the current study was to evaluate the extent of microfiber exposure, with particular attention being paid to cellulosic natural/artificial textile fibers, in *M. barbatus* and *M. merluccius* exemplars from the Adriatic Sea (Central Mediterranean Sea), considering (a) the economic importance of these species [20,21], (b) their role as ecotoxicological bioindicators in the Mediterranean Sea [17,22], (c) the limited available data on natural/artificial microfiber exposure [22,23], despite the fact that they may be harmful to exposed organisms [24], and (d) the heterogeneous spatial distribution of anthropogenic debris in the Mediterranean Sea [9,25]. The sampling site was chosen by taking into account that (a) although this basin accounts for only 5% of the total area of the Mediterranean Sea, several stocks of commercially important fish species come from this semi-enclosed sea [26]; (b) the Adriatic Sea represents a preferential site in the Mediterranean Sea in terms of the input of several pollutants from the land, also due to the low circulation of water [2]. A monitoring strategy based on multiple species with different characteristics [27] was applied to investigate microfiber pollution, and the isolated particles were identified by the use of a visual approach [8,18,28,29], coupled with Fourier-transform infrared (FTIR) analyses.

## 2. Materials and Methods

### 2.1. Materials

Sodium chloride (ACS grade), hydrogen peroxide solution at 30% (ACS grade), and potassium hydroxide (ACS grade) were provided by Carlo Erba (Val de Reuil, France). For the filtration of digested samples and solutions, cellulose nitrate (pore size 8 µm) and acetate (pore size 0.45 µm) filters (Sartorius Stedim Biotech, Göttingen, Germany) were used, respectively. The filtrating system was supplied by Advantec (Dublin, CA, USA). Macroporous silicon filters with a 5 μm pore size (MakroPor) were purchased from Thermo Fisher Scientific (Waltham, MA, USA).

### 2.2. Fish Sampling

A total of 100 fish samples from 2 commercial species (*M. barbatus* = 50, *M. merluccius* = 50) intended for human consumption were collected from local fishermen after landing them in the Molise Region, Italy. The fishes come from the Central Mediterranean sub-region, within sub-area FAO 37.2.1 of the FAO-GFCM (General Fisheries Commission for the Mediterranean), which includes the Adriatic Sea. The selection of the sampling period (autumn–winter) was based on previous findings that showed increased microfiber exposure related to the spawning season and weather conditions [19]. Specimens of similar length were sorted and, after ascertaining skin integrity, the selected samples were individually wrapped in aluminum foil and transported to the laboratory. The total length (cm) and body weight (g) were recorded for each specimen (Table 1), and then they were stored at −20 °C until further laboratory analysis. Each sample was labeled with an identification code.

In order to evaluate the correlation between the fish health status and microfiber ingestion, Fulton’s condition factor (K) was determined, using the following formula: K = 100 × (weight/total length^3^) [28,30].

### 2.3. Microfiber Extraction and Quantification

The fish were thawed at room temperature and washed with previously filtered water. The stomach and intestine were removed and weighed, avoiding damaging the organs and not losing any of their contents. The GITs were individually transferred into a glass Erlenmeyer flask and the extraction protocol used by Santonicola et al. [19], which was previously validated [29,31], was applied. Briefly, the microfiber extraction was based on a digestion step using a 10% potassium hydroxide (KOH) solution and incubation at 45 °C overnight, followed by density-flotation separation using a NaCl hypersaline solution (density 1.2 g/cm^3^) and filtration. To remove tissue residues, a 15% H_2_O_2_ solution was added to the membranes, which were stored in an oven at 45 °C overnight. The extracted particles were microscopically observed using a stereomicroscope (M205C, Leica, Wetzlar, Germany) at a magnification of 0.78–16×, and micrographs of each microfiber were collected in order to categorize the microfibers according to size classes (ImageJ software, release 1.43, NIH, Bethesda, MD, USA), colors (blue, black, transparent/clear, purple, sky blue, pink, red, and orange) and morphological features [19]. The microfiber count was obtained by analyzing each micrograph. One blank control, without any tissue, was treated in parallel with each batch of 10 samples, and the microfiber count identified in the blanks was directly subtracted by reducing the number of microfibers corresponding to each sample. The blank samples exhibited a number of 4 ± 2 microfibers.

### 2.4. Microfiber Identification

The optical micrographs were analyzed by two different operators in order to discriminate between synthetic and natural/artificial microfibers according to certain morphological characteristics, such as cross-sections, breakages, and the appearance of the fiber body and ends [8,19,28,32,33], using as a reference the micrographs of microfibers of known origin [29]. Visual identification has been proven to be a useful screening method in order to reduce the number of microfibers in the spectroscopic analyses [8,28], taking into account the analytical issues that may hamper microfiber identification using spectroscopic techniques [18].

Therefore, to confirm microfiber identification based on their morphological features, their chemical composition was evaluated by FTIR microscopy. FTIR microscopy was performed on a subset of isolated microfibers. In detail, 4 filters containing microfibers representative of each morphological pattern (natural cellulosic microfibers, twisted like flat ribbons with frayed edges, regenerated shaped microfibers of a regular diameter and with a striated surface, and synthetic rigid microfibers with a cylindrical cross-section and smooth surface) [19], isolated from both species, were selected. Microfibers were transferred to MakroPor silicon filters (Thermo Fisher Scientific, Waltham, MA, USA) using distilled water and analyzed using an FTIR microscope (Nicolet iN10 MX, Thermo Fisher Scientific, Waltham, MA, USA). FTIR spectra were acquired in transmission mode, averaging 64 scans, with a resolution of 4 cm^−1^, and then the acquired spectra were matched with the FTIR library of the device to define the polymer type. OMNIC™ Specta 2.0 software was used for an analysis of the acquired spectra and a match rate higher than 70% was considered useful for microfiber identification.

A total of 26 microfibers were subjected to FTIR analyses, and 15 microfibers were identified with a certainty of over 70%. The data used to calculate microfiber abundance, size, and color distribution were based on visual identification.

### 2.5. Contamination Precautions

Special care was taken to prevent sample contamination during sample dissection, microfiber extraction, sorting, and identification. Extraction procedures were carried out in a clean room with limited access by staff. Samples were covered with aluminum foil at all times and exposed only during dissection, which was performed under a clean laminar flow hood. Before filtration, each filter was examined under the microscope to detect microfiber contamination. Cotton laboratory coats and nitrile gloves were worn during the analysis. Distilled water and all the solutions were filtered through cellulose acetate membranes (pore size 0.45 μm) before use. The material and working surfaces were cleaned three times with filtered water before use and between samples. After filtration, membranes were kept in Petri dishes that were new and unopened, previously rinsed with prefiltered water.

### 2.6. Statistical Analysis

Statistical analysis was carried out using IBM^®^ SPSS^®^ Statistics software, version 23.0 (IBM, Chicago, IL, USA). The data were tested for normality using a Shapiro–Wilk test and for homogeneity of variance using Levene’s test. A non-parametric Kruskal–Wallis test was also performed. Indeed, all the data analyzed did not comply with the assumption of homogeneity of variances. A Pearson correlation test was performed to assess any significant correlations. A 5% significance level was used for all statistical tests.

## 3. Results

### 3.1. Microfibers in Fish

Overall, 100 fishes (*M. barbatus* = 50 and *M. merluccius* = 50) were analyzed in this study, in line with the guidelines of the Marine Strategy Framework Directive [34], which recommends a sample of at least 50 specimens of similar size per species to represent the population [35].

Microfibers were successfully isolated from the GIT and then recovered on filter surfaces. The observation of these filters under the optical microscope allowed us to evaluate that a total of 36 (72%) red mullet and 34 (68%) European hake sample fishes had ingested microfibers. The average number of microfibers per GIT of each individual and per gram wet weight (w.w.) of fish tissue and GIT are reported in Table 2.

In total, 38% of the samples of *M. barbatus* showed a microfiber abundance of 1–4 particles/GIT, while 22% and 10% of samples exhibited microfiber levels of 6–15 and 20–27 particles/GIT, respectively; only one sample showed a total of 42 particles. Regarding *M. merluccius*, 28% of the samples had ingested 1–4 particles/GIT, 30% showed a microfiber abundance of 5–19 particles/GIT, while 8% exhibited microfiber levels of 24–34 particles/GIT, and one sample showed a total of 67 microfibers.

The number of microfibers/g w.w. per individual differs between species (Kruskal–Wallis test, *p =* 0.009), but no correlation between fish biometric values and fiber number was evident (Pearson correlation, *p* > 0.05—see Tables S1–S4). However, despite these results, a trend could be observed. Larger red mullet specimens contained more microfibers/g w.w. per individual, while the number of microfibers/g w.w. per individual increased in the hake small exemplars. The evaluation of Fulton’s condition factor showed that the samples with low K values ingested more microfibers, but the correlation was not statistically significant (Pearson correlation, *p* > 0.05—see Tables S5 and S6).

**Figure 1:**
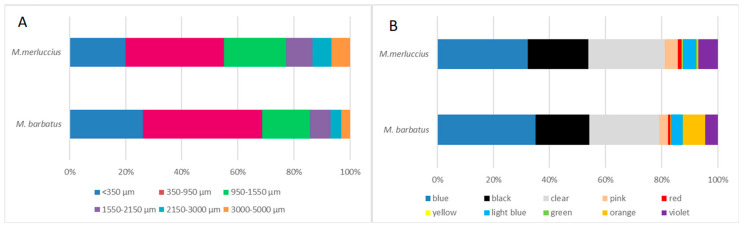
Size (**A**) and color (**B**) distribution of the microfibers detected in fish samples from the Adriatic Sea.

In both species, natural/artificial microfibers, classified according to their morphological features, were the most numerous among the isolated microfibers (*M. barbatus*: 78%; *M. merluccius*: 82%). Concerning microfiber length, the values ranged from 83.99 to 4606.38 µm, and the prevalent size class was 350–950 µm (*M. barbatus*: 42.5%; *M. merluccius*: 35.2%; see Figure 1A). Natural microfibers showed a mean length of 865.26 and 1100.07 μm in red mullet and hake, respectively, while the average length of synthetic microfibers was 962.49 μm in red mullet and 1150.37 μm in hake samples.

Most of the fibers were blue (*M. barbatus*: 35%; *M. merluccius*: 32.17%), followed by clear (*M. barbatus*: 25%; *M. merluccius*: 27.25%) and black (*M. barbatus*: 19.1%; *M. merluccius*: 21.72%), and other colors (Figure 1B).

### 3.2. Microfiber Characterization

**Figure 2:**
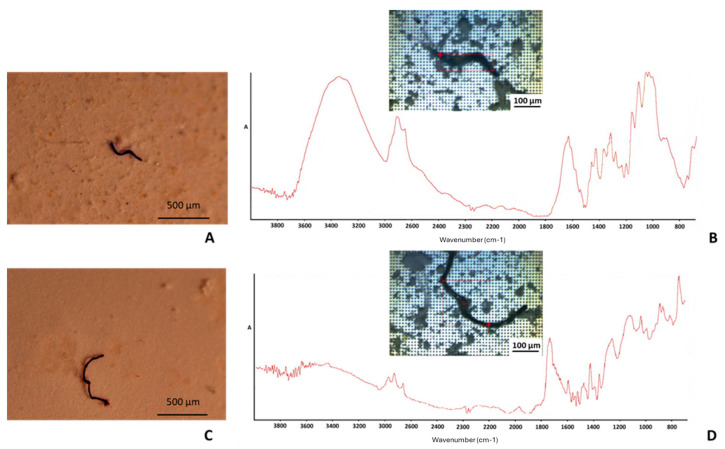
Optical micrographs and FTIR spectra of microfiber particles identified as: (**A**,**B**) cellulose and (**C**,**D**) polyester.

A subsample of microfibers was carefully isolated and identified through micro-FTIR analysis of no. 4 filters of samples taken from both species (Figure 2).

The absorption bands corresponding to the main functional groups of cellulose and polyester microfibers were identified by matching the acquired spectra with the FTIR spectral database, using Thermo Scientific OMNIC™ Spectra™ 2.0 software. In detail, the main absorption bands identified in the FTIR spectra of cellulose and polyester microfibers [24] are reported in Table 3.

Cellulose was the predominant type of material found, making up 92.8% of the sub-sample in the FTIR assay (cellulose 50%, cotton 35.7%, and rayon 7.14%). Natural and semisynthetic forms of cellulose are hard to distinguish between in FTIR assays [9]; therefore, they were grouped together [19]. In contrast, the presence of synthetic polymers was significantly less marked, with polyester as the prevalent type. The identification of the chemical composition of a subset of microfibers confirmed the prevalence of natural/artificial cellulosic microfibers.

## 4. Discussion

The Mediterranean Sea is an important hotspot for marine litter accumulation, with high spatial heterogeneity levels and distribution [36]. In this context, the Adriatic Sea (Central Mediterranean), due to the low circulation of water, is characterized by significant microplastic concentrations, including large numbers of microfibers (85%), which is also linked to the lack of suitable waste management facilities [37].

The occurrence of anthropogenic microfibers in the marine environment has recently grown in terms of evidence, showing that most of these particles are non-synthetic fibers [9]. However, while different investigations have revealed the dominance of synthetic microfibers within the GIT of marine organisms [3,12,13,14], little attention was paid to the extent of natural and artificial microfiber ingestion in biota [5].

Results showed that red mullets and hakes from the Adriatic Sea seem to be easily exposed to microfiber ingestion, due to the frequent presence of these elements within their GITs (*M. barbatus* 72% and *M. merluccius* 68%), confirming the wide distribution of microfibers in the Adriatic semi-enclosed basin. Moreover, microfiber identification highlighted the prevalence of cellulosic fibers (almost 80% of all extracted particles), which may thus represent a consistent fraction of the total fibers in this area [2].

*M. barbatus* is extensively used for monitoring marine pollution because they are heavily exposed to microdebris while living within a benthic environment. In addition, *M. merluccius* has been designated as a sentinel species for its trophic links between pelagic and demersal habitats [27]. Both species are the most important commercial species for bottom trawlers in Italy and are employed in different traditional dishes, also as a whole, and marketed (in the case of hakes) as processed fish products under important brand names [38]. The microfiber exposure of the red mullets collected from different areas of the Mediterranean Sea [2,13,17,19,23,28,39,40,41] has been widely reported, but only a few studies refer to natural/artificial microfiber contamination [2,13,19,28]. Similarly, for European hakes, different studies have investigated microfiber exposure as a part of microplastic contamination in the Mediterranean Sea [2,17,22,23,39,42,43], but the occurrence of natural microfibers was reported in only a limited number of surveys [2,19,43]. Therefore, in order to make the comparison with the literature more consistent, only those studies that included natural/artificial microfibers were considered (Table 4).

Regarding red mullet, our results are comparable with those obtained by Avio et al. [2], despite the lower frequency of ingestion detected in the current survey. In contrast, the percentage of hake samples containing microfibers was similar to those detected by Avio et al. [2], but our results showed higher levels of contamination. Comparing the results obtained with red mullet specimens from the Tyrrhenian Sea [19], a higher number of contaminated samples and lower microfiber levels were detected. In contrast, a higher frequency of ingestion but lower levels of contamination were recorded in the Adriatic hakes. This confirms the heterogeneous distribution of microlitter in Mediterranean waters [36].

In line with our results, all the studies agreed with the dominance of natural/artificial cellulosic microfibers among the ingested microdebris, except for that of Capillo et al. [13]. This evidence further corroborates that the evaluation of microfiber pollution should include natural/artificial and synthetic fibers [2,19].

The occurrence of numerous fibers in larger specimens of red mullet could be related to accidental ingestion during the suction capture of small prey that lives on the sea floor, in addition to those derived from the ingestion of contaminated prey [44], while adult hakes may be mostly exposed by eating prey that contains microfibers [45]. However, despite species-specific differences, similar microfiber characteristics (color and length distribution) were observed. In both species, most of the isolated microfibers were blue (33.6%), clear (26.1%), and black (20.3%). From the available literature, these microfiber colors were found to be the most abundant in Mediterranean waters [46], and the high rates of ingestion of particles of these colors may also reflect their availability along the Adriatic trophic web [2]. Moreover, considering that the median microfiber length detected in Mediterranean seawater was 1.33 mm [9], the fiber size distribution, detected in the current study and in previous surveys [28,43], in *M. barbatus* and *M. merluccius* inhabiting this area confirms their role as bioindicators of marine pollution [27], and that the types and characteristics of fibers inside the fish are closely related to those found in the water samples [9].

Considering the wide occurrence of microfibers and fibrous microplastics in different environmental compartments and organisms [15,47], a more environmentally friendly textile industry should be promoted by governments by developing sustainable approaches and advanced solutions to mitigate microfiber pollution in the seas and oceans [48] and the consequent exposure of marine biota, in particular with respect to species intended for human consumption. Secondly, the interrelationship of environmental health with that of animals and humans should be addressed, focusing attention also on the potential transfer of microfibers through the food chain with detrimental consequences for the consumers [4]. The occurrence of microfibers in the edible parts of fish and processed seafood was documented, but more data are needed to make a safety risk assessment [4,49]. In the current survey, the occurrence of microfibers was assessed in the GITs of commercial fish species. The consumer could be exposed to microfibers when eating whole fish or due to the translocation of these particles from the digestive tract to other organs and tissues, in addition to the contamination of edible parts that may occur during fish handling and preparation [16]. Therefore, microfiber contamination should be investigated along the entire fish supply chain in order to understand the additional sources of contamination, including airborne fallout from clothing and machinery [50], during the processing and packaging of fishery products.

## 5. Conclusions

In this study, the microfiber exposure of two commercially important fish species in the Mediterranean was investigated showing that natural/artificial cellulosic microfibers are dominant among the ingested debris.

Considering the role of *M. barbatus* and *M. merluccius* as bioindicators of marine litter pollution, the obtained results highlighted the contamination of the Adriatic Basin, pointing to the need for the implementation of mitigation measures to decrease microfibers entering the marine ecosystem and the consequent exposure of marine biota. Regarding their importance as fishery resources, the frequency of ingestion and the microfiber levels detected in the analyzed samples proved the importance of focusing more attention on natural/artificial microfiber contamination, which is often insufficiently considered. The development of harmonized analytical methods could help obtain comparable results among the different studies and adequately evaluate the extent and the impact of microfiber pollution on the marine food web. Further studies are needed in order to understand the implications of microfiber ingestion on fish health conditions, with a particular focus on reproductive function, and the mechanisms of translocation of these particles in different organs and tissues, including fish edible parts. In light of these findings, the entire fish supply chain should be monitored to better identify the risks and implications for consumer health.

## Figures and Tables

**Table 1:** Biometric data related to *M. barbatus* and *M. merluccius* specimens (average ± SD).

	Mean Length (cm) ± SD	Mean Weight (g) ± SD	Mean GIT Weight (g) ± SD
*M. barbatus* (50)	10.96 ± 0.58	25.27 ± 5.07	1.25 ± 0.23
*M. merluccius* (50)	18.64 ± 1.16	58.85 ± 10.93	1.68 ± 0.93

**Table 2:** Summary of microfiber levels in fish samples from the Adriatic Sea.

	*M. barbatus*			*M. merluccius*		
	Mean ± SD	Median	Range	Mean ± SD	Median	Range
Number of MFs/GIT in all individuals examined	5.90 ± 8.84	2.00	-	6.98 ± 12.10	2.00	-
Number of MFs/GIT in individuals containing MFs	8.19 ± 9.62	3.50	1–42	10.26 ± 13.52	6.50	1–67
Number of MFs/g w.w. of GIT	4.65 ± 6.57	1.42	0.66–28	5.02 ± 8.17	1.11	0.40–36.25
Number of MFs/g w.w. of individual	0.23 ± 0.33 ^a^	0.07	0.02–1.45	0.12 ± 0.22 ^a^	0.02	0.01–1.17

Letters correspond to significant differences among the data within the groups (Kruskal–Wallis test, *p* = 0.009).

**Table 3:** FTIR absorption band assignment for polyester and cellulose microfibers.

Wavenumber (cm^−1^)	Assignment
Polyester	
1714	C=O stretching
1447	aromatic ring of C=C
1249	C–O–C stretching
1092	O=C–O–C stretching
1014	O=C–O–C stretching
720	heterocyclic aromatic ring
Cellulose	
3600 to 3200	OH-stretching
2918	CH_2_ asymmetrical stretching
2850	CH_2_ symmetrical stretching
1735	C=O stretching
1638	amide I
1422	CH_2_ scissoring
1150	Anti-symmetrical C-O-C stretching
1100	anti-symmetric C-O-C in-plane stretching
1057	C-O-C in-plane stretching
1030	C-O stretch

**Table 4:** Microfiber abundance in *M. barbatus* and *M. merluccius* from the Mediterranean Sea.

Number of Samples	Sampling Area	Frequency of Ingestion	Average Number of Microfibers/GIT	% of Natural/Artificial Microfibers	References
50 *M. barbatus*	Adriatic Sea	72%	5.9	78%	Current study
8 *M. barbatus*	Adriatic Sea	100%	5.37	>80%	[2]
21 *M. barbatus*	Tyrrhenian Sea	14.28%	0.3	-	[13]
118 *M. barbatus*	Catalan coast	50%	1.48	57%	[28]
50 *M. barbatus*	Tyrrhenian Sea	62%	8.3	58%	[19]
50 *M. merluccius*	Adriatic Sea	68%	6.9	82%	Current study
10 *M. merluccius*	Adriatic Sea	60%	2	>80%	[2]
82 *M. merluccius*	Catalan coast	66%	1.39	77.8%	[43]
50 *M. merluccius*	Tyrrhenian Sea	72%	8.9	68%	[19]

## Data Availability

The data that supports the findings of this study are available from the corresponding author [M.C.], upon reasonable request.

## Supplementary Materials

The following supporting information can be downloaded at: https://www.mdpi.com/article/10.3390/foods14071237/s1, Table S1: Pearson correlation between the number of MFs and *M. barbatus* size (expressed in g w.w.); Table S2: Pearson correlation between the number of MFs and *M. barbatus* size (expressed in cm); Table S3: Pearson correlation between the number of MFs and *M. merluccius* size (expressed in g w.w.); Table S4: Pearson correlation between the number of MFs and *M. merluccius* size (expressed in cm); Table S5: Pearson correlation between the Fulton factor and MFs/g w.w. in *M. barbatus*; Table S6: Pearson correlation between the Fulton factor and MFs/g w.w. in *M. merluccius.*

## Abbreviations

The following abbreviations are used in this manuscript:

GIT	Gastrointestinal tract
FAO-GFCM	General Fisheries Commission for the Mediterranean
