# Supplementary material for: Natural and Regenerated Cellulosic Microfibers Dominate Anthropogenic Particles Ingested by Commercial Fish Species from the Adriatic Sea
Source: Foods. 2025 Apr 1;14(7):1237. doi: 10.3390/foods14071237 (PMC11988341; doi:10.3390/foods14071237)
Supplement: Supplementary file 1 [file foods-14-01237-s001.zip › foods-3538952-supplementary.pdf]

Supplementary information

Table S1. Pearson correlation between the number of MFs and *M. barbatus* size (expressed in g w.w.).

Correlations

		Weight	MFs
Weight	Pearson Correlation	1	0.062
	Sig. (2-tailed)		0.670
	N	50	50
MF	Pearson Correlation	0.062	1
	Sig. (2-tailed)	0.670	
	N	50	50

Table S2. Pearson correlation between the number of MFs and *M. barbatus* size (expressed in cm).

Correlations

		MFs	Length
MFs	Pearson Correlation	1	0.181
	Sig. (2-tailed)		0.209
	N	50	50
Length	Pearson Correlation	0.181	1
	Sig. (2-tailed)	0.209	
	N	50	50

Table S3. Pearson correlation between the number of MFs and *M. merluccius* size (expressed in g w.w.).

Correlations

		MFs	Weight
MFs	Pearson Correlation	1	-0.150
	Sig. (2-tailed)		0.297
	N	84	50
Weight	Pearson Correlation	-0.150	1
	Sig. (2-tailed)	0.297	
	N	50	50

Table S4. Pearson correlation between the number of MFs and *M. merluccius* size (expressed in cm).

Correlations

		MFs	Length
MFs	Pearson Correlation	1	-0.051
	Sig. (2-tailed)		0.725
	N	84	50

Length	Pearson Correlation	-0.051	1
	Sig. (2-tailed)	0.725	
	N	50	50

Table S5. Pearson correlation between the Fulton factor (K) and MFs/g w.w. in *M. barbatus.*

Correlations

		K	MF/g w.w
K	Pearson Correlation	1	-0.193
	Sig. (2-tailed)		0.179
	N	50	50
MF/g w.w.	Pearson Correlation	-0.193	1
	Sig. (2-tailed)	0.179	
	N	50	50

Table S6. Pearson correlation between the Fulton factor (K) and MFs/g w.w. in *M. merluccius.*

Correlations

		K	MF/g w.w
K	Pearson Correlation	1	-0.131
	Sig. (2-tailed)		0.363
	N	50	50
MF/g w.w.	Pearson Correlation	-0.131	1
	Sig. (2-tailed)	0.363	
	N	50	50
